# Impact of Micronutrient Supplementation on Pesticide Residual, Acetylcholinesterase Activity, and Oxidative Stress Among Farm Children Exposed to Pesticides

**DOI:** 10.3389/fpubh.2022.872125

**Published:** 2022-06-14

**Authors:** Srujana Medithi, Yogeswar Dayal Kasa, Vijay Radhakrishna Kankipati, Venkaiah Kodali, Babban Jee, Padmaja R. Jonnalagadda

**Affiliations:** ^1^Symbiosis Institute of Health Sciences, Symbiosis International (Deemed) University, Pune, India; ^2^Food Safety Division, Indian Council of Medical Research – National Institute of Nutrition, Hyderabad, India; ^3^National Institute of Nutrition-TATA Centre for Excellence in Public Health Nutrition, Indian Council of Medical Research – National Institute of Nutrition, Hyderabad, India; ^4^Biostatics Division, Indian Council of Medical Research – National Institute of Nutrition, Hyderabad, India; ^5^Department of Health Research, Ministry of Health and Family Welfare, Government of India, New Delhi, India

**Keywords:** pesticide exposure, micronutrient status, vitamin-minerals supplementation, oxidative stress, farm children

## Abstract

The present interventional study aimed to assess the impact of micronutrient supplementation on pesticide-residues concentrations, vitamins, minerals, acetylcholinesterase activity and oxidative stress among 129 farm children (9–12 years, *n* = 66 and 13–15 years, *n* = 63) involved in farming activities in Ranga Reddy district, Telangana, India. Our data showed the presence of five organophosphorus pesticide residues (chlorpyrifos, diazinon, malathion, monocrotophos, and phosalone) among children before-supplementation (both age-groups); while post-supplementation, only two pesticide residues (chlorpyrifos and diazinon) were detected indicating improved metabolic rate. Vitamin E, copper, magnesium and zinc levels were also improved in both the age-groups and manganese levels were significantly increased only among children of 13–15 years age group. Further, post-supplementation also showed an improvement in acetylcholinesterase activity and a decrease in lipid peroxidation among both the age groups of children. However, further research for ascertaining the ameliorating effect of micronutrients in preventing adverse effects of organophosphorus pesticides must be conducted.

## Introduction

Farmers are at higher risk of pesticide exposure during farming activities (1). However, the nutritional status of an individual also plays a significant role in mitigating the possible susceptibility to pesticide exposure which can be addressed with proper supplementation which may result not only in the improvement of overall health status but also in providing increased protection against exposure. Moreover, the relationship between adverse effects of pesticide exposure and nutritional deficiencies may be a threatening signal from public health perspective (2).

Further, pesticides also induce oxidative stress leading to the generation of free radicals thereby causing alterations in the body's antioxidant or oxygen-free radical-scavenging enzyme system. Oxidative stress can be defined as the imbalance between the production of free radicals capable of causing possible peroxidation in the cells and the body's antioxidant defense by vitamins and minerals, which are the structural components of many essential enzymes involved in metabolism. They also act as the second line of defense in the human body to ameliorate the pesticide induced oxidative stress (3). A series of studies showed that exogenous supplementation of vitamins and minerals improve various biochemical parameters including the levels of serum lipid peroxide Glutathione transferase (GST) and Superoxide Dismutase (SOD), catalase (CAT), serum zinc, copper of farmers and also capable to reverse the adverse effects imposed by pesticides (1, 4, 5). A study conducted among the sprayers in grape gardens (Maharashtra, India) reported a significant decrease in serum lipid peroxide, GST and increased levels of RBC-SOD, RBC-catalase, serum zinc and copper after 15 days of supplementation (1, 5). Further, it also concluded that Vitamin E plays crucial role in restoring the antioxidant enzymes such as SOD, CAT, and CP among the population exposed to pesticides upon micronutrient supplementation. Similar studies in human erythrocytes also showed the potential protective effects of vitamins C and E pre-treatment and notably reversible effects by the treatment (4). Additionally, supplementation studies in animals have shown the protective effects of vitamin A, E, and zinc against pesticide induced toxicity (6–10).

The susceptibility to pesticide exposure depends not only on nutritional status but also on physiological factors like age and gender (11). Children working in the farms are more prone to exposure to toxins in proportion to their respective body weights and thus, are more vulnerable than adults to possible consequences of adverse effects of pesticide exposure (12–14). They receive greater doses of pesticides and metabolize toxicants slower than adults as their metabolic processes are immature and as their immune system may be weak there by making them more susceptible and hence are incapable of detoxifying and excreting the pesticides by retaining the residues in their body for a longer period and therefore, the exposure may sometimes be higher than that of adults (15, 16). Further, the exposure will also be more, if their parents are engaged in farming and if they live are in close proximity to treated farmlands as they directly in contact with the soil containing the persistent pesticide residues causing adverse health effects even at very low levels (17, 18). Two behavioral traits associated with children's exposure to pesticides include their hand to mouth ingestion, which may increase their toxic chemical intake in dust or soil and playing in or close to the treated soils resulting in their increased exposure to pesticides (19). Studies suggest that older children also help their parents in farming activities and get exposed to pesticides (20). It has been demonstrated that developing infants or children present “windows of vulnerability” that determine a higher response to toxic effects than at later stages of life (21) and hence, are at a greater risk as they eat, drink and breathe more than adults.

Further, pesticides exert toxicity by inhibiting the acetylcholinesterase (AChE) enzyme which degrades acetylcholine (ACh), an essential neurotransmitter in the central nervous system (CNS). AChE activity in children has been also associated with measurable physiologic changes. In epidemiological studies, a single measure of AChE activity in children is considered as an adequate indicator of pesticide exposure (22). Lower AChE activity was associated with overall lower neurobehavioral development, primarily affecting attention, inhibitory control and memory among boys exposed to organophosphate pesticides. Thus, AChE inhibition is an indicator of exposure and other neurotoxic mechanisms (23). However, studies among the pesticide exposed children and oxidative stress are meager. Further, the studies assessing the effects of micronutrient supplementation (which involves the provision of single or multiple micronutrients in the form of capsules, tablets, drops, or syrup) on pesticide toxicity among humans particularly children are meager. Hence, the present study aimed to assess the impact of intervention with micronutrients on pesticide residual, AChE activity and oxidative stress among farm children exposed to pesticides.

## Materials and Methods

### Study Area and Participants

The present study was conducted among farm children belonging to Ranga Reddy district (Telangana state, India), consisting of 26 villages, which have the highest area under cultivation for cotton crops which involves the highest consumption of pesticides (24). The other major crops cultivated are rice, maize, jowar, pulses, and vegetables. Based on the survey, Shankarpalli mandal in Ranga Reddy district was found to have been cultivating majorly the cotton and other vegetable crops. The subjects involved in farming activities were randomly selected from two villages (Maharajpet and Mahalingapuram) to include them in the present study and was conducted in accordance with the Helsinki Declaration (Ethical Principles for Medical Research Involving Human Subjects).

The sarpanch (village head) of the selected villages was approached to identify the target population. The inclusion criteria considered were that the children were of the age groups 9–12 and 13–15 years old who were helping their parents and participating in various agricultural activities in their own farms. However, the children who never participated in agricultural activities and were known to suffer from any kind of chronic illness or any physical disability were exempted from the present study. Subsequently, the subjects fulfilling the inclusion criteria were randomly selected and were contacted through their parents to explain about the purpose of the study. Further, after obtaining their consent and upon willingness to participate, a pre-tested questionnaire was administered to them (*n* = 378) to collect information on socio-demographic particulars, and working schedule in the agricultural fields. Based upon the responses provided by them, a total of 129 children who were helping their parents and who so ever were involved in farming activities were found eligible to be included in the study. The identified children were categorized into two age groups; 9–12 years (*n* = 66) and 13–15 years (*n* = 63), who were assumed to provide any clinical significance due to the difference in their growth/developmental stages.

### Study Design

**Figure 1:**
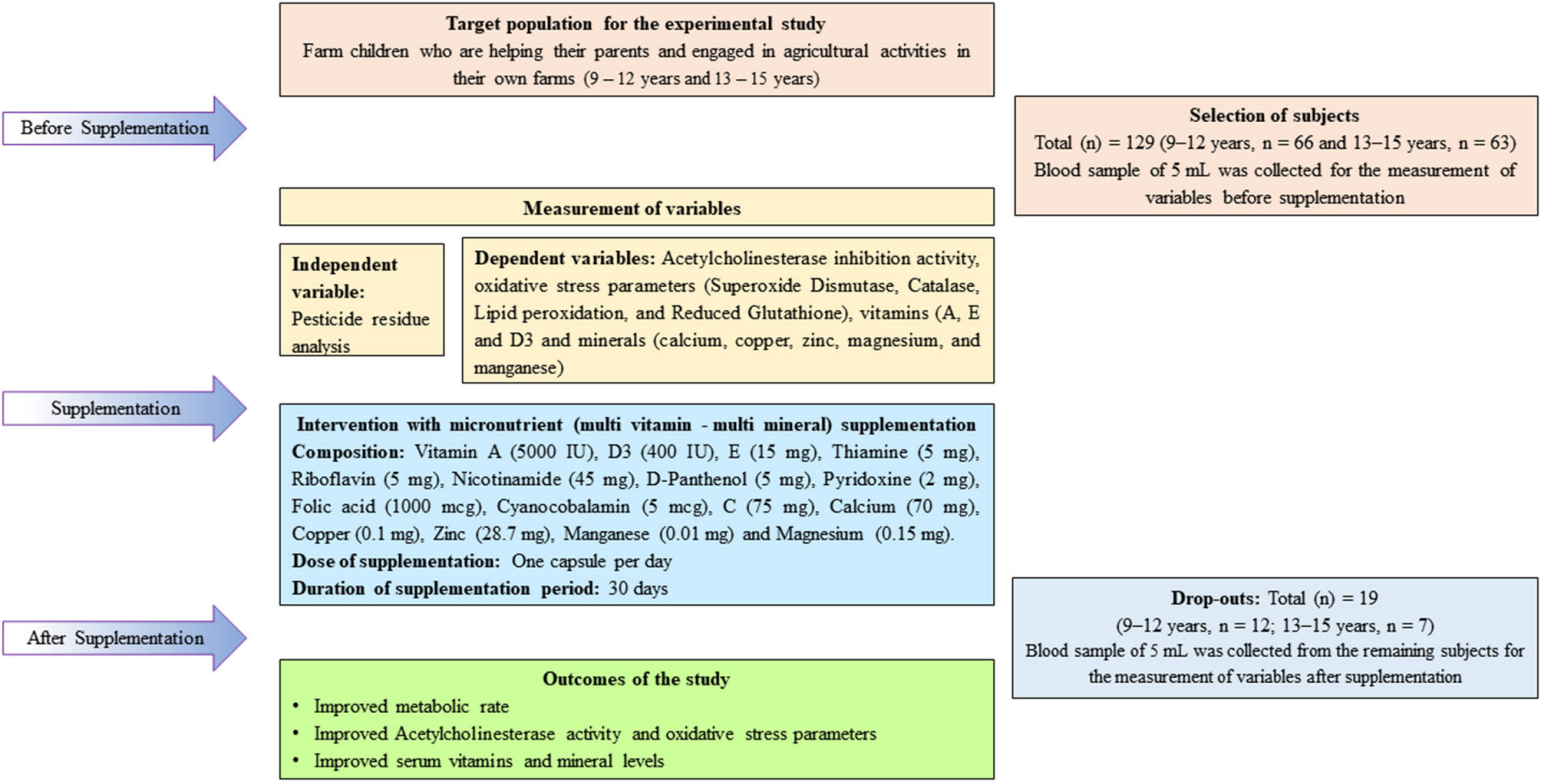
Conceptual framework of the study.

The present study commenced with collecting baseline data from the study subjects. The anthropometric data was measured and blood samples (5 mL) were collected from both the age group of children, 9–12 years (*n* = 66) and 13–15 years (*n* = 63). Subsequently, these children were provided with micronutrient supplementation (multi vitamin—multi mineral) capsules for 30 days. The details of baseline assessment, composition/dose of the intervention, drop-outs, and the intervention sampling are provided in Figure 1. At the end of the intervention period, there were about 19 (9–12 years, *n* = 12 and 13–15 years, *n* = 7) drop-outs of children, since they did not follow the advocacy of supplementation as per the guidelines provided to them. Therefore, blood samples (5 mL) collected from children were from 9 to 12 years (*n* = 54) and 13–15 years (*n* = 56) of age group after the supplementation, for the analysis of various biochemical parameters which are indicated as above to observe the effects of supplementation/post-intervention if any.

### Measurements

#### Anthropometric Measurements

The body weights of the subjects (without footwear) were taken using a digital weighing machine while their heights were measured using the portable anthropometric rod and the Body Mass Index (BMI) was calculated using Quetelet's index (kg/m^2^). The BMI-for-age was calculated by referring to the BMI Percentile Calculator for Child and Teen provided by the Centers for Disease Control and Prevention (CDC), U.S. Department of Health and Human Services.

#### Blood Sample Collection

Blood samples (5 mL) were collected through venipuncture in Becton Dickinson (BD) vacutainer tubes under aseptic conditions from each study subject at baseline and after supplementation. The samples collected were transported in thermal boxes containing gel packs (maintained at −10°C) from the field to the laboratory and were subjected to centrifugation to separate the serum/plasma and stored at −80°C until further analyses to estimate pesticide residue levels, AChE levels, oxidative stress parameters (SOD, CAT, lipid peroxidation, and reduced glutathione), and also the micronutrient status (by measuring the levels of vitamins A, E, and D3 and minerals: calcium, copper, zinc, magnesium, and manganese).

#### Method Development and Validation for Organophosphorus Pesticide Residues Analysis

Nine organophosphorus pesticides (chlorpyrifos, diazinon, dichlorvos, dimethoate, malathion, monocrotophos, phorate, phosalone, and quinalphos) which were predominantly used in the study area were analyzed in the serum samples. Briefly, the extraction procedure is as follows. The serum samples (0.5 mL) were extracted in a 5 mL glass vial. The samples were spiked with 50 μL of TPP (concentration 50 ng/mL) and after thorough vortexing (for 2 min), the samples were loaded onto conditioned SPE cartridges. The cartridges were pre-conditioned using 500 μL of 0.25% formic acid, 1 mL acetonitrile (twice) and 1 mL of distilled water (twice). Later, the cartridges were subjected to washing using 1 mL of distilled water (twice) and 1 mL of 10% methanol (once). The elution was done using 1 mL of acetonitrile and the eluents were collected in a glass test tube. The tubes were subjected to evaporation under dry nitrogen (at 50°C for about 45 min or until dry). The residues were then reconstituted using acetonitrile (200 μL) and subjected to centrifugation (at 2,000 rpm, 5 min) using a refrigerated centrifuge. The final extract was collected in the vials to be injected into Liquid Chromatography-Tandem Mass Spectrometry (LC-MS/MS) system (Shimadzu LC 20AD, Applied Biosystems MDS Sciex 4000-Q TRAP triple quadrupole) and auto-sampler (SIL-HTC model) controlled using Analyst software (version 4.1.2). Zorbax SB-C18 HPLC Column (4.6 × 150 × 5 μm) was used to carry out the chromatographic separation. The analysis was carried out in Multiple Reaction Monitoring (MRM) positive turbo ion spray (ESI) mode with high resolution. The flow was maintained in a gradient mode using two mobile phases (mobile phase A–80:20 v/v of acetonitrile: water containing 0.5% formic acid and mobile phase B–95:5 v/v of acetonitrile: water containing 0.5% formic acid) which were maintained at a flow rate of 0.3 mL per min. The total run time of the analysis was 32 min and the sample injection volume was set to 35 μL. The ion-spray voltage (IS) of 5,500 eV and interface heater at a temperature of 500°C were maintained.

The method was validated for linearity, the limit of detection (LOD), limit of quantification (LOQ), and inter/intra-day validations. The linearity of the standards was tested by spiking the solvent using the standard solutions in the range of 0.2–750 ng/mL (0.2, 0.5, 1, 2, 5, 10, 20, 50, 100, 200, 500, and 750 ng/mL). A linearity curve was plotted against the concentration of the analyte vs. a ratio of the peak area of the analyte and the peak area of the internal standard. As the sample is a complex matrix, the analysis was carried out using a matrix matched calibration solution such as blood samples from human volunteers (*n* = 28). The collected samples were tested for the presence of pesticide residues with the method that was developed for the present study. Only those samples that were free from pesticides to be studied were considered for the validation of this method. The samples were then subjected to extraction using SPE cartridges. The calibration curve obtained with the concentration range of 0.2–750 ng/mL was found to be linear with regression square constant (*r*^2^) > 0.99 (ranged between 0.990 and 0.999). Quality control for the method was also done using the human serum samples (spiked at a concentration ranging from 15, 150, and 350 ng/mL along with the internal standards). The analytes were extracted using the SPE cartridge method as described earlier. The sensitivity of the method was evaluated by determining the limit of detection (LOD) of all pesticides (0.5 ng/mL) and the limit of quantification (LOQ) (1 ng/mL) except for Phorate (2 ng/mL). The inter-day and intra-day precision of the method was calculated by running six replicate samples spiked with stock solutions of analyte standard solution at 15, 150, and 350 ng/mL concentrations and the internal standard. The percentage accuracy of inter/intra-day ranged from 92.7 to 107.3% and 93.9 to 104.2%, respectively. The variation coefficients of intra/inter-day precision ranged from 0.3 to 11.7% and from 2.5 to 8.1%, respectively. The average recoveries ranged between 90.4 and 105.8% for the most commonly used pesticides in the present study.

#### Estimation of Vitamins A and E Levels

Vitamins A and E play a pivotal role in the antioxidant protective mechanism. Vitamin A is essential to quench the singlet molecular oxygen activity, while the active form of vitamin E can act directly on various oxygen radicals, thereby combating oxidative stress (25, 26). Therefore, the active forms of vitamins A and E (viz., retinol and tocopherol, respectively) were measured in the plasma samples of the subjects in the present study. The plasma samples were processed by using 300 uL of plasma (along with internal standards: 50 μL of retinyl acetate and tocopherol acetate at a concentration of 50 μg/dL and 1.5 mg/dL, respectively) in 4 mL polypropylene tube to which a mixture of 1 mL of Acetonitrile: Ethyl acetate (50:50) were added and mixed vigorously (5 min) using vortex followed by centrifugation (5,000 rpm, 5 min) and the supernatant was collected. Only 0.8 mL of it was used for evaporation under nitrogen at 50°C (15 min). The concentrated extract was re-constituted using 100 uL of methanol and was mixed thoroughly for about 30 s using vortex and was transferred to High-performance liquid chromatography (HPLC) [Shimadzu LC 20AD equipped with SIL-HTC Shimadzu autosampler, Photon diode array detector (PDA) controlled by LC solutions software]. Zorbax SB-C18 HPLC Column (4.6 × 150 × 5 μm) was used for the analysis. The mobile phase was prepared by using 600 mL of acetonitrile, 350 mL of methanol and 50 mL of dichloromethane in 1 liter bottle. The mixture was sonicated for about 15 min for degassing. A constant flow rate (1 mL/min) was maintained with an injection volume of 40 μL. The retention time for retinol and tocopherol was found to be 2.7 and 7.6 min, respectively. The analysis for Vitamins A (retinol) and E (α-tocopherol) was done at 325 and 292 nm wavelengths, respectively.

#### Estimation of 25-Hydroxy Vitamin D [25 (OH) D] Levels

Vitamin D has been considered as a non-enzymatic antioxidant compound and therefore, may play a vital role in antioxidant defense in the body (27). Hence, in the present study, the active form, i.e., vitamin D3 was analyzed in the serum samples of the subjects. Vitamin D3 levels in the serum samples were analyzed following the instructions of the 25(OH) Vitamin D ELISA kit (Calbiotech Inc., USA), as well as the protocol (28). The analysis was conducted using 10 μL of serum sample and was performed in replicates. The absorbance was measured at 450 nm using a multi-well plate reader (Biotek Synergy H1 Hybrid, Gen5 2.01 software). The kits were validated for their performance characteristics such as Sensitivity – 0.71 ng/mL; Intra-Assay Precision – Mean – 24.7 ng/mL, SD – 1.7, CV – 5.4%; Inter-Assay Precision – Mean – 25.7 ng/mL, SD – 2.1, CV – 4.9%.

#### Estimation of Mineral Levels

The minerals such as copper, zinc, manganese and magnesium are considered as vital components of the endogenous antioxidant enzyme system as they act as catalytic co-factors, while calcium plays a significant role in regulating the optimal activity of vitamin D (29, 30). Hence, studying the levels of these minerals is also essential and therefore, their levels were analyzed in the serum samples of the subjects. About 300 μL of serum sample was taken and extracted using 3 mL of nitric acid and 1.5 mL of hydrogen peroxide in the digestor tubes and subjected to microwave digestion using CEM—Mars 5 (One-touch technology) microwave digestor. The solutions obtained after digestion were further analyzed using Atomic Absorption Spectroscopy (AAS) (Perkin Elmer AAnalyst 400 with Software WinLab, 32 version) with suitable lamps. A seven-point calibration was run for each test element from highest to lowest concentrations for minerals viz., calcium, copper, zinc, manganese, and magnesium to plot the calibration curve. The instrument was then set to concentration mode and the unknown samples were aspirated to obtain their respective concentration from the calibration curve (31).

#### Determination of Acetylcholinesterase Inhibition Activity

Exposure to organophosphorus pesticides causes inhibition of AchE activity, resulting in the accumulation of acetylcholine at the nerve endings, leading to cholinergic hyperactivity and therefore, AchE activity has been considered as a reliable biomarker (32). The AchE activity was determined using a cholinesterase (kinetic method) kit (Reckon Diagnostics Pvt. Ltd., India) and also as per the methods mentioned previously (33). About 20 μL of unknown serum sample was used for the estimation and the activity was measured using a Semi-auto analyser (Merck Microlab 300).

#### Determination of Oxidative Stress Markers

The reactive oxygen species (ROS) formed during the pesticide induced oxidative stress may affect the primary defense mechanism such as SOD, CAT and GSH and also capable to induce lipid peroxidation leading to the generation of secondary oxidation products (malondialdehyde-MDA) (34). Therefore, they were considered as indicators of oxidative stress in the blood samples to be measured (35). They are the SOD, measured using the commercially available Superoxide Dismutase Assay kit (Cayman Chemicals Pvt. Ltd., USA) and following the procedures published earlier (36). The readings were taken at 460 nm using the micro-well plate reader (Biotek Synergy H1 Hybrid, Gen5 2.01 software) to obtain the unknown concentrations. While, CAT activity was assayed spectrophotometrically (Shimadzu-UV-1800, UV Probe 2.42 version software) by the method prescribed earlier (37). Further, the total protein (Biuret method) was assayed following the kit method (38) (Reckon Diagnostics Pvt. Ltd., India) and semi-auto analyzer to obtain the unknown concentrations. Lipid peroxidation (by measuring levels of malondialdehyde, MDA) in the plasma was quantified as thiobarbituric acid reactive substances (TBARS) by spectrophotometric method (39). Reduced glutathione (GSH) levels were measured using the fluorimetric assay using Jasco FP-6500 Spectrofluorometer (Spectra manager software) (40).

### Statistical Analysis

The database was prepared with its codes after its entry into the computer. Statistical analysis was performed using SPSS 21.0 version software. The percentage of samples detected with pesticide residues (both the age groups) among subjects before and after the supplementation was calculated (9–12 years, *n* = 66 and 13–15 years, *n* = 63). While the quantitative results of the levels of pesticide residues in the serum samples among the subjects were given as Mean and standard deviation (SD). Further, the quantitative results of levels of micronutrients, AChE activity and oxidative stress parameters were expressed as Mean and SD. Paired Student's *t*-test was performed among the subjects to compare the difference if any, between “before” and “after” supplementation and the statistical significance was considered at *p* < 0.05 and *p* < 0.01.

### Ethical Statement

The study protocol was approved by the Institutional Ethical Committee (IEC), Indian Council of Medical Research (ICMR)—National Institute of Nutrition (NIN), Hyderabad (Approval No. 3/2013/I).

### Consent of the Subjects for Participation and Publication

The informed consent sheets (in the local language—Telugu) were provided to the parents of the study subjects and also explained the study's purpose. Further, written consent was obtained from the children's parents who were willing to participate in the study to use the same for study purposes and subsequently publish as a research paper in peer-reviewed journal while protecting their identity.

## Results

### Demographic Particulars

A total of 129 children (9–12 years, *n* = 66 with a mean age of 10.5 and 13–15 years, *n* = 63 with a mean age of 13.9 years) who were selected for the present study were administered with a questionnaire to obtain the details of their working schedule and their major involvement in farming activities in the agricultural fields (Table 1). The mean BMI of the 9–12-year group children was 14.3 while it was 15.6 for the 13–15 years age group children and placing the BMI-for-age at the 1st percentile and 2nd percentile, respectively, under the underweight category.

**Table 1:** Demographic particulars.

	**9–12 years**	**13–15 years**
	**(*n* = 66)**	**(*n* = 63)**
**Mean age (in years)**	10.5	13.9
**Working schedule (%)**		
During weekends	86	97
Before going to school	82	83
After returning from school	77	90
**Major activities in the agricultural field (%)**		
Preparation of soil	77	92
Sowing	82	95
Watering	83	90
Cutting	97	97
**Major activities during spraying operation (%)**
Lifting the pesticide tank	92	94
Filling the tank with water	97	87
Mixing of formulations	67	97
Washing the tank after use	91	90

### Levels of Pesticide Residues Before and After Supplementation

Residues of five organophosphorus pesticides viz., chlorpyrifos, diazinon, malathion, monocrotophos, and phosalone were detected in the children of both the age groups before the micronutrient supplementation, while only two pesticide residues such as chlorpyrifos and diazinon were detected after the supplementation (both age group**s**) (Table 2).

**Table 2:** Levels of pesticide residues detected in the serum samples of children aged 9–12 years and 13–15 years before and after supplementation.

**Pesticide**	**Before supplementation**			**After supplementation**		
	**(*****n*** **=** **66)**			**(*****n*** **=** **54)**		
	**Percentage of samples detected with residues**	**Mean (ng/mL)**	**Standard Deviation**	**Percentage of samples detected with residues**	**Mean (ng/mL)**	**Standard Deviation**
**Among 9–12 years children**						
Chlorpyrifos	94	0.57	0.07	24.07	0.72	0.13
Diazinon	10.6	0.53	0.03	3.7	0.59	0.02
Malathion	68	0.82	0.6	ND	NA	NA
Monocrotophos	27	0.54	0.04	ND	NA	NA
Phosalone	18	0.54	0.02	ND	NA	NA
**Among 13–15 years children**						
Chlorpyrifos	90	0.81	0.3	19.6	0.77	0.12
Diazinon	41	0.57	0.09	1.7	0.51	NA
Malathion	20	0.53	0.03	ND	NA	NA
Monocrotophos	19	0.75	0.4	ND	NA	NA
Phosalone	32	1.08	0.4	ND	NA	NA

*ND, Not Detected; NA, Not Applicable*.

### Impact of Micronutrient Supplementation on the Levels of Vitamins and Minerals

**Figure 2:**
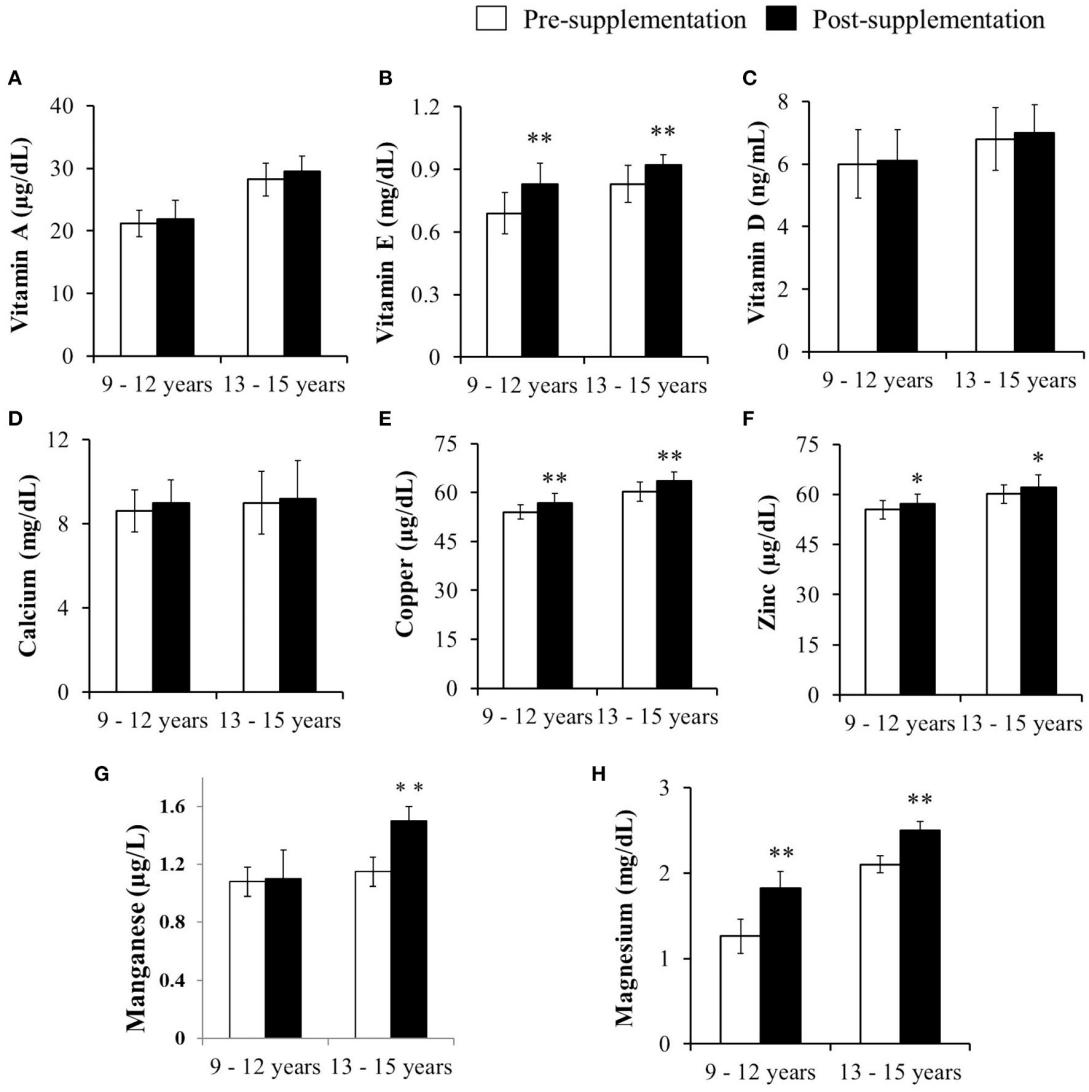
Impact of micronutrient supplementation on the vitamins and mineral levels among the pre- and post-supplemented children of 9–12 and 13–15 years age group. Results are expressed as mean and SD values of vitamin and mineral levels – **(A)** Vitamin A, **(B)** Vitamin E, **(C)** Vitamin D, **(D)** Calcium, **(E)** Copper, **(F)** Zinc, **(G)** Manganese and **(H)** Magnesium in the blood samples of pre-supplemented (*n* = 54) and post-supplemented (*n* = 54) children of 9–12 years and also pre-supplemented (*n* = 56) and post-supplemented (*n* = 56) children of 13–15 years age groups. Data was analyzed by Paired Student's “t”-test. Significance at *p* < 0.05 and *p* < 0.01 was denoted as * and ** respectively.

There observed an increase in vitamins A and D3 and calcium levels among the children of both the age groups after supplementation; however, this increase was not statistically significant. On the other hand, levels of vitamin E and minerals viz., copper, magnesium and zinc were increased significantly in both the age groups on supplementation (*p* < 0.01 and *p* < 0.05). Further, the manganese levels were also significantly increased but their increase was observed only among the children of 13–15 years age group (*p* < 0.01; Figure 2).

### Impact of Micronutrient Supplementation on AChE Activity and Oxidative Stress Parameters

**Figure 3:**
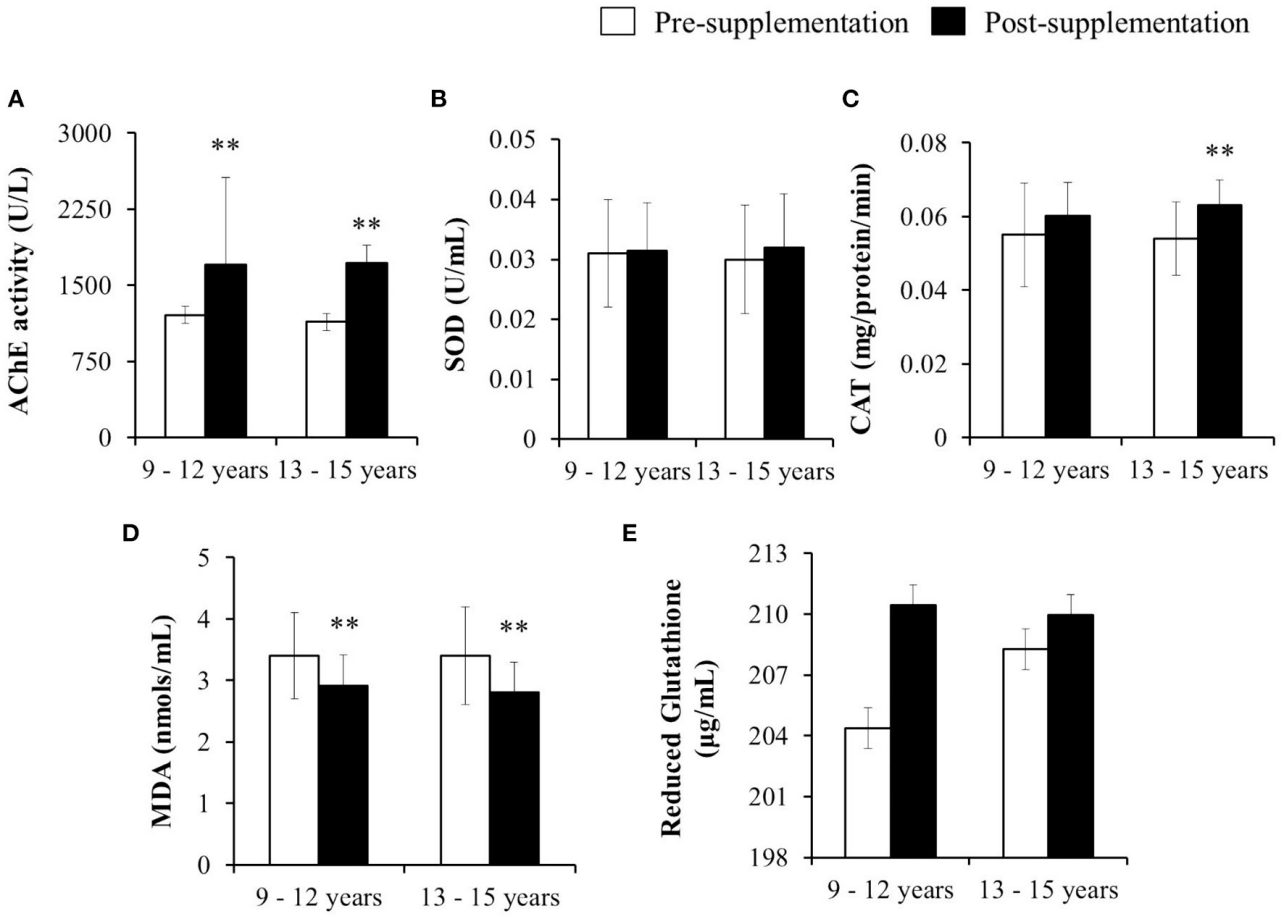
Impact of micronutrient supplementation on the AChE activity and oxidative stress parameters among the pre and post-supplemented children of 9–12 and 13–15 years age group. Results are expressed as mean and SD values of AChE and oxidative stress parameters – **(A)** AChE activity, **(B)** SOD, **(C)** CAT, **(D)** MDA and **(E)** Reduced Glutathione in the blood samples of pre-supplemented (*n* = 54) and post-supplemented (*n* = 54) children of 9–12 years and also pre-supplemented (*n* = 56) and post-supplemented (*n* = 56) children of 13–15 years age groups. Data was analyzed by Paired Student's “t”-test. Significance at *p* < 0.01 was denoted as **.

A statistically significant improvement in the AChE activity and a decrease in lipid peroxidation was observed upon the supplementation among both the age groups of children (Figures 3A,D). While CAT levels were also improved after supplementation in both the age groups of children but the increase was significant only among the children of the 13–15 years age group (*p* < 0.01; Figure 3C). As regards SOD and reduced glutathione levels, though an increase was observed in both the age groups of children but it was not statistically significant (Figures 3B,E).

## Discussion

In the present study, it was observed that the blood pesticide residue levels were lower after supplementation. To the best of our knowledge, there has been no study reporting an association between micronutrient supplementation and the detectable pesticide residue concentrations in the blood and among the children of two different age groups studied in the present investigation. It appears possible that the micronutrient supplementation could have influenced/enhanced the pesticide metabolism, there by facilitated to escalate the elimination of residues from their bodies due to which, they were either not detected or decreased to the lowest level of detection in the serum samples. Nevertheless, two residues viz., chlorpyrifos and diazinon were detected in serum samples even after supplementation indicating the retention of these residues in the body could either be due to their slower metabolism and/or poor/lesser excretion suggesting the need for further investigations with long term follow up and supplementation to substantiate the findings.

Furthermore, a significant increase was also seen in vitamin E levels and minerals such as copper, zinc, magnesium among subjects of both the age groups, upon supplementation, whereas the manganese levels were siginificantly higher compared to original levels prior to the supplementation among 13–15 years age group children. It is a known fact that minerals such as copper, zinc, manganese and magnesium play an important role in the antioxidant metalloenzymes that are considered not only as critical components of the endogenous antioxidant enzyme system but also as catalytic co-factor for SOD and CAT (29). While some like copper plays a pivotal role in the electron transfer reactions because of its unique redox cycling ability, manganese to enhance oxidative stress resistance by replacing the more reactive iron cofactor in certain iron-containing enzymes susceptible to oxidative attack (41). Further, the minerals such as zinc and magnesium are not only involved in the synthesis and functioning of glutathione, while magnesium is also involved in lipid peroxidation and malondialdehyde formation (42). In the present study, it was observed that the protection provided by the minerals upon their supplementation among the study subjects exposed to organophosphate pesticides by their decreased levels in the serum and with significant increase of minerals after supplementation. It is evident from the previous study that the vitamins in the biological system not only suggest their pathways and also the beneficial effects of nutrition adequacy and possible adverse health effects due to their deficiency (43). Vitamin A is essentially important in many biological processes and possesses the ability to quench the singlet molecular oxygen activity in scavenging the free-radicals generated in the body to combat oxidative stress (25). In addition, vitamin D which not only functions as vitamin but also yet times functions as hormone will be affected, if exposed to pesticides as the pesticides are known to be endocrine disruptors (44). Of late, it is also considered as a non-enzymatic antioxidant compound (27). In the present study, it is pertinent to note that in the present study the subjects were supplemented with micronutrients after determining the five organophosphate pesticide residues on day zero (pre-supplementation) and the subjects were continued to involve themselves in the farming activities (exposure to organophosphate pesticides) for the subsequent 30 days (post-supplementation) found to have shown an increase in the levels of vitamin D3 among the children of both the age groups upon supplementation. Interestingly, the levels of Vitamin E have also significantly increased among the children of both the age groups indicating the protection provided by tocopherol in regulating the ROS and hence found a decrease in the oxidative stress parameters like lipid peroxidation induced due to pesticide exposure. This shows that the levels have increased after 30 days of supplementation. It was further found the supplementation found to have been effective in restoring the inherent antioxidant system associated with free radical damage (26).

In the present investigation, significantly lower levels of AChE were found among the children of both the age groups suggesting that the intervention with micronutrients played an important role in improving the AChE activity. Similar observations were found in earlier studies conducted elsewhere among the children exposed to organophosphate pesticides. Further, the studies have also reported the significantly decreased cholinesterase (<6,334 U/L) levels among the children playing in orchard farms treated with organophosphate pesticides in India (45). Some other studies conducted in Egypt have also reported the decreased levels of cholinesterase among the applicators in age group 9–18 years of age group as compared to the controls (46). Another study conducted in Egypt with eight time points (from day 0 to 269) among adolescent (age group 12–21 years) pesticide applicators (predominantly Chlorpyrifos) reported a significant depression of AChE activity (at two points, day 146 and 269) relative to non-applicators (47). A similar study reported that repeated occupational exposure to Chlorpyrifos is an important determinant of neurological symptoms in adolescent applicators of 12–21 years age group (48). Another study carried out among adolescents (aged 12–21 years) working in agricultural farms in Egypt assessed a four-time point in pesticide application season and observed a significant inhibition of AChE during Chlorpyrifos application at single point of time (49). A similar study conducted among Indian sprayers in grape gardens reported a significant decrease in serum lipid peroxide, GST and increased levels of SOD, CAT, serum zinc and copper after 15 days of supplementation (1, 5). The reported incidence of such studies concluded that Vitamin E plays a crucial role in restoring the antioxidant enzymes such as SOD, CAT and CP among the population exposed to pesticides (1, 5). Vitamin E (α-tocopherol), the major lipid-soluble antioxidant present in all cellular membranes, protects against lipid peroxidation and has the potential to break the radical chain and directly act on a variety of oxygen radicals and peroxides. It is proven that vitamin E, is not only capable of regulating the generation of superoxide and other ROS, but also found to be most effective in restoring the inherent antioxidant system, protecting cell internal structures against free radical damage and acts as an antioxidant for cellular membrane lipids (50). Furthermore, in the present study, decreased lipid peroxide level in the serum may be accredited to vitamin C, E, Zinc, and Copper supplementation in the form of capsules to the children of both the age groups. Thus, the present study has demonstrated the beneficial role of micronutrient supplementation in improving the AChE activity and as well reducing the oxidative stress parameters in children exposed to pesticides.

The current study findings suggested that supplementation with micronutrients could be a simple interventional strategy in reducing the pesticide-induced susceptibility among the farm children. Overall, it was further observed from the present study, that micronutrient supplementation appears to tune-up metabolic rate in children as it facilitates to escalate the excretion of the residues in the serum among the children suggesting a protective effect from adverse effects of exposure. Further, the intervention with micronutrient supplementation would also help attain an optimal nutritional status which would further help in improving their susceptibility toward organophosphate pesticide exposure. However, further, an elaborated, detailed research with a larger sample size on prospective intervention studies among the farmers (various physiological age/gender) is needed to gain a comprehensive understanding of the possible ameliorating effects of micronutrients on the adverse effects of pesticide exposure. It is further recommended that the children should be guided appropriately in not allowing them to use the empty containers of pesticides as play items to minimize the exposure. However, the chronic inadvertent pesticide exposure which is likely to occur in agricultural farms/households can also be minimized by creating awareness among them through extensive training and education programs. Therefore, it is further recommended that the farm children should be made available with nutritious food to the extent possible to combat the likely possible inadvertent exposure while working in the agricultural fields of their own.

## Data Availability Statement

The raw data supporting the conclusions of this article will be made available by the authors, without undue reservation.

## Ethics Statement

The studies involving human participants were reviewed and approved by Institutional Ethical Committee (IEC), Indian Council of Medical Research (ICMR)—National Institute of Nutrition (NIN), Hyderabad (Approval No. 3/2013/I). Written informed consent to participate in this study was provided by the participants' legal guardian/next of kin.

## Author Contributions

SM was responsible for conceptualization, data collection, statistical data analysis, and writing. YK was involved in data collection. VRK supervised the intervention program. VK provided the guidance for data analysis and interpretation. BJ was involved in reviewing the first and final draft of the manuscript and provided the critical inputs. PJ was the supervisor of the project and involved in the editing and reviewing of the manuscript. All authors approved the version to be published.

## Funding

This work was funded by the Indian Council of Medical Research (ICMR), Government of India, India (GIA/45/2014-DHR).

## Conflict of Interest

The authors declare that the research was conducted in the absence of any commercial or financial relationships that could be construed as a potential conflict of interest.

## Publisher's Note

All claims expressed in this article are solely those of the authors and do not necessarily represent those of their affiliated organizations, or those of the publisher, the editors and the reviewers. Any product that may be evaluated in this article, or claim that may be made by its manufacturer, is not guaranteed or endorsed by the publisher.
